# Knowledge Concerning Dietary Supplements among General Public

**DOI:** 10.1155/2019/9629531

**Published:** 2019-06-24

**Authors:** Gabriela Kołodziej, Barbara Cyran-Grzebyk, Joanna Majewska, Krzysztof Kołodziej

**Affiliations:** Institute of Physiotherapy, Faculty of Medicine, University of Rzeszow, Poland

## Abstract

*Introduction. *Diet and physical activity are widely recognized in maintaining health and preventing diseases. Currently, dietary supplements are also commonly believed to play a role in health promotion. However, a dietary supplement lacks single uniform definition within the scope of legal or nutritional sciences. Data on the usage of dietary supplements in Europe are still limited. The aim of this study was to assess subjective and objective knowledge of the inhabitants of south-eastern Poland about diet and supplementation in sport.* Materials and Methods. *The research was conducted in the south-east of Poland between October 2017 and July 2018 during three events organized by the University of Rzeszów. Initially, the study covered 500 people. During the preliminary analysis, 90 people were excluded from the study because they did not meet the previously well-explained rules of conduct during the study or did not meet the eligibility criteria for the tests. Finally, 410 people were qualified for the study. The survey method was a questionnaire with single-answer option.* Results. *Our study revealed statistically significant subjective and objective level of knowledge about diet and supplementation in terms of sex. Also, a correlation was found between sex and the fact of being involved in physical activity, as well as the subjects' knowledge of diet and supplementation in sport.* Conclusion. *High prevalence of dietary supplement use was found in our study population, but still significant percentage of the study group demonstrate inaccurate information about these products. Hence, there is an urgent need to provide the community with education and access to scientific and unbiased information.

## 1. Introduction

Diet and physical activity are well recognized to play important role in maintaining health and disease prevention [1]. Regular physical activity reduces the risk of cardiovascular disease, some types of cancer, osteoporosis, diabetes, obesity, high blood pressure, depression, stress, and anxiety [2]. Currently, taking dietary supplements (DS) is also commonly believed to be health promoting activity. However, DS lack single uniform definition within the scope of legal or nutritional sciences. The European Food Safety Authority (EFSA) defines food supplements as concentrated sources of nutrients or other substances with a nutritional or physiological effect intended to supplement a normal diet [3]. These products may contain vitamins, minerals, herbs, amino acids and other substances, or their constituents [4]. DS are usually offered in tablets, capsules, powders, or pills. DS intake intends to prevent disease, upgrade mental and general health, enhance sport performance, and compensate for dietary deficiencies [5]. However, the exact benefits of DS consumption are still not well established [6]. The alarming fact is that a considerable number of people consuming DS do not seek any medical advice before taking them [7].

According to current regulations, DS are considered dietary products and therefore they are available not only in pharmacies, but also in other places, such as groceries or online stores. Vast number of people do not tell the difference between medication and DS frequently administering different groups of preparations at the same time neglecting possible interactions between the substances contained in them [8].

According to the studies, about half of the adult US population uses some form of DS, and a similar prevalence is likely to occur in many other countries despite economic, regional, and cultural differences [9].

In the USA, the most commonly used DS include vitamins, minerals, and plant products, while in the EU countries, products containing vitamins and minerals account for 50% of total sales, DS containing other substances have 43% market share, and energy drinks are the least popular (7%) [10].

Data on DS use in Europe are still limited; the main data are available from commercial market analysis rather than consumer surveys. Therefore, the aim of this study was to investigate the current knowledge of DS among Polish society and to explore the need for education in this area.

This research has been presented at the conference XI International Days of Rehabilitation Needs and Standards of Rehabilitation in Rzeszów, Poland, on 28th February–1st March 2019 and published as a part of post-conference materials.

## 2. Material and Methods

The study was carried out in the south-eastern Poland between October 2017 and July 2018 during the three events organized by the University of Rzeszów. Initially, the study covered 500 people. During the preliminary analysis, 90 people were excluded from the study because they did not meet the previously well-explained rules of conduct during the study or did not meet the eligibility criteria for the tests. The inclusion criteria were as follows: healthy individual (without cognitive impairment and other diseases), aged 18-40 years, and informed written consent to participate in the study. Exclusion criteria included giving multiple answers in the questionnaire, deleting an answer and providing a different one, not answering any of the questions, and lack of informed written consent to participate in the survey. Finally, 410 people were qualified for the study. The characteristics of the study group are detailed in Table 1.

The study group covered 410 respondents including 250 woman and 160 men. In terms of Body Mass Index, 83.17% the respondents had normal weight, 9.27% were overweight, 5.86% were underweight, and only 1.70% of study group were obese. 60.00% of the study group were urban residents, while 40.00% of the respondents lived in a rural area. Most of the subjects did not undertake any physical activity and 47.56% actively engaged in physical activity. Table 1 presents the details of the study population.

## 3. Statistical Methods

Qualitative variables were expressed by means of quantity and indicators of the structure. The Pearson chi-square test was used to compare parameters between the studied subgroups. Statistical analysis was performed using the STATISTICA 13.1. The result of the statistical test was the test probability (p), with small values indicating the statistical significance of the considered dependence. We assumed in the paper that

1. p <0.05 means a statistically significant relationship,
2. p <0.01 means a statistically highly significant relationship,
3. p <0.001 means a statistically very highly significant relationship.

## 4. Results

The data from Table 2 suggest statistically significant dependence between the sex of the studied population and their knowledge about the diet and supplementation in sport. Most of the subjects did not know what kind of DS was included in group A. The correct answer is that the bicarbonate or citrate were included in group A of DS. Research shows that only 29.20% of the studied women knew the correct answer, while 45.00% of men answered correctly. The correct answer to the question about the percentage of the total caloric value of the protein in the meal was given by 46.25% of men in the study group and only 22.40% of woman. Similar observation was made in another question, about the percentage of the total calorific value of carbohydrates and fats in a meal. The studied group of men had a higher level of knowledge than woman. Generally, the level of knowledge about DS was extremely low. The respondents assessed their subjective level of knowledge about diet and supplementation in sport as a low (n= 167; p≤0.001). Table 2 presents the above-mentioned observations.

We found statistically significant relationships between the subjects who engaged in physical activity and their knowledge about the supplements included in group A, the percentage of the total calorific value of carbohydrates and fats in a meal. People who engaged in physical activity had better knowledge about diet and supplementation in sport than people who did not engage in any physical activity. Our research indicated that more than half (52.48%) of the studied population engaged in physical activity and did not know the definition of supplementation. The respondents engaging in physical activity assessed their subjective level of knowledge about diet and supplementation in sport as a low (p≤0.001). Similarly, the study group who were not engaged in physical activity stated the same. Detailed information is provided in Table 3.

On the basis of the research included in the table, it has been stated that there is a statistically significant relationship between the studied population who used DS and their knowledge about supplementation in sport.  Table 4 shows that 58.59% of study population did not use DS, but they knew the definition of supplementation. On the other hand, many of the studied population did use DS but they did not know its definition (73.38%). We found statistically significant relationships between the studied population who used DS and the kind of supplements included in groups A, B, C, and the percentage of the protein, carbohydrates, and fats in meal. Only 35.36% of the study group knew what kind of substances is included in group A of DS. Statistical significance appeared also at the subjective level of knowledge about diet and supplementation in sport. In this case we observed that respondents rated their knowledge at a low level. Detailed information is included in Table 4.

We found statistically significant relationships between the age and training experience and population who used DS. Our research shows that 65.63% of studied population were people who did not use DS, aged 19-24 years old. Similarly, the largest group among those who used dietary supplements were people aged 19-24. The smaller group of population who did not use DS were people in the age range 25-30 years, and in population who used DS the smaller group was older than 31 years. The statistical significance was shown in the training experience too. Most of the studied population who used DS had training experience of 6-11 years, and in the group who did not use any DS, 71.88% had training experience of 1-5 years. The detailed data are presented in Table 5.

Based on the data included in the table, it has been stated that there is a statistically significant relationship between the studied population who engaged in physical activity and subjective and objective knowledge about caloric demand per kilogram of body weight in sport of different character. 55.28% of the studied population who did engage in physical activity did not use DS. The correct answer to the question about caloric demand per kilogram of body weight in endurance sport is 70 kcal/kg of body weight. Only 26.70% of the studied population in the group who did engage in physical activity answered correctly. Similarly, the same group answered correctly in the following two questions about the caloric demand per kilogram of body mass in high-speed sports and caloric demand per kilogram of body weight in strength sports. Our research shows that the studied population who did engage in physical activity know better than population who did not engage in physical activity about the caloric demand per kilogram of body weight in sport with different character. The knowledge about the caloric demand in both of these groups can be assessed as little (on a low level; p≤0.001). The same data confirms Table 6.

On the basis of the research included in the table, it has been stated that there is a statistically significant relationship between the training experience and the knowledge about diet and supplementation in sport. Our research showed that people with the longest training experience had better knowledge about what kind of substances were included in group A of DS, 45.95%.  Table 7 shows that the studied population with the training experience of 6-11 years had better knowledge about what kind of substances were included in group C of DS in sport and the percentage of the total caloric value of the protein and carbohydrates in the meal, accordingly 44.27% and 50.00%. On the basis of the research included in the table, it has been stated that there is a statistically significant relationship between the training experience and subjective knowledge about diet and supplementation in sport. The group with the shortest training experience, 45.81%, had average knowledge about supplementation. The vast knowledge was declared by the group with 6-11 years training experience, 48.36%. The group with the longest training experience, 45.95%, had little knowledge. Detailed information is included in Table 7.

We found statistically significant relationship between the age and the objective and subjective level of knowledge about diet and supplementation in sport. 59.22% of the studied population aged over 31 knew the definition of supplementation in sport (p**≤**0.00). Statistical significance also appeared in the question concerning knowledge about what kind of supplements were included in groups A, B, and C; greater knowledge about this issue was demonstrated by the group in the age range of 25-30 years.  Table 8 shows that the group aged 25-30 years answered correctly to the other questions about knowledge of diet and supplementation in sport. 55.71% of the studied group aged 25-30 had large knowledge about this subject. The group aged over 31 years had the least knowledge.

## 5. Discussion

It has become a popular trend in recent years to take care of a perfect figure and an athletic body. It is common knowledge that not only physical activity but also a properly balanced diet helps to achieve these goals. Active working life does not always allow one to prepare proper nutritious meals. In recent years, one might have noticed a growing interest in DS. It is especially noticeable among active people doing various forms of sport and recreation [11]. An increase in the use of DS among the Polish can be observed not only among athletes, but also among children, adolescents, and pregnant women [12]. It is obvious that physically active people show significantly increased demand for energy and some nutrients, e.g., protein, carbohydrates, and vitamins, especially combined with intense physical activity [12, 13].

Many researchers wonder why athletes use DS, and not solely a balanced diet. Doing sport forces us to limit the food consumption, while simultaneously enforcing additional supplementation to balance the supply of nutrients [14]. Maughan et al. published examples of the use of supplements, i.e., on the micronutrient deficiencies management, providing convenient forms of energy and macroelements, and providing direct or indirect benefits for the results, such as supporting an intensive training process [15].

In our research conducted with the help of the author's questionnaire, over half of the respondents were women (n = 250). 83.17% of the studied group had normal BMI and 52.44% of the respondents declared that they did not engage in any physical activity. After the initial analysis of the results, the majority of people were unable to give the correct definition of dietary supplementation. Men more often responded correctly to the question about supplements from group A. Overall, the knowledge assessment level was low. Manore M, Patton-Lopez M, Meng Y et al. also undertook the assessment of the knowledge level about supplementation in sport among adolescents aged 14-18 using a questionnaire with regard to place of residence, sport activity, diet habits, etc. 45.60% of people achieved a positive result in terms of the assessed knowledge. Among the study group, similarly to our study, men gave more correct answers and showed a higher level of knowledge about the possibilities of supporting sport activity with diet and supplementation. Young athletes should be subjected to education in the area of the studied subject, in order to avoid side effects of DS on the body in the future [16].

The results acquired in the studies confirm the popularity of using DS among people regardless of whether they engage in sport activities or not. The majority of the respondents in the conducted research did not declare practicing physical activity, and the knowledge level about supplementation was rated subjectively low. People reporting regular physical activity more often selected correct answers in regard to supplements from groups A, B, C in comparison with physically inactive people. The analysis of the results showed that physically active people had a problem with giving the correct definition of dietary supplementation, which contradicts the universal statement that it is athletes and physically active people who are most often interested in supplementation. Zdešar Kotnik et al. in the published article presented an analysis of the popularity of using DS. Their use turned out to be widespread among teenagers (69%), athletes (76%), and physically inactive people (66%). Definitely more dietary supplementation is widespread among men who have legitimized their use in order to improve athletic performance and enhance muscle development and function. Women also use supplements, but to a smaller degree. They emphasize mainly the role of DS in improving the immune system. The longer time of regular sport activity correlated with the higher frequency of supplements use, especially among men who were athletes in team games. A high percentage of teenagers (41%) decided to use a supplement on their own without prior advice from parents or other people (30%) [17].

Similar results were acquired in our research considering the correlation of the knowledge assessment of physically active and inactive people, and the knowledge level within the range of the caloric requirements from protein and carbohydrates. Similar results were acquired in both groups. The presented results show that it does not matter whether the respondents have or have not used supplements, and their knowledge about the caloric requirements was very little. Couture. et al. conducted a study among trainers, whose aim was to check their knowledge in the area of sports nutrition and nutrition practices. 47 high school coaches filled out a questionnaire on nutritional knowledge and practices. Less than 30% of coaches answered correctly the general nutritional questions about carbohydrates and lipids. There was no statistically significant difference in nutrition knowledge between coaches in team and individual sports, and between men and women. However, the respondents with higher education ranked higher than others (73.3% against 63.3%, p <0.05). Coaches who had the authorization to exercise their profession also achieved better results than those who did not have a coaching certificate. The authors also showed that the most popular source of information on nutrition used by the coaches was the Internet on the level of 55%. The most popular dietary practices recommended by the coaches to improve athletes' performance were hydration and eating food rich in protein. Recommendations regarding the use of DS were extremely rare and were suggested only to football coaches, strength sports [18].

Analyzing the results of our research in terms of the period of time of practicing the sport, people with longer record had better knowledge of diet and supplementation. Similar observations were published by Hull et al. The authors showed that athletes had greater knowledge of supplementation and nutrition in comparison with the remaining population filling the survey [19].

The authors also noted the difference in the level of knowledge with regard to sex [19]. In our research, the vast majority of the interviewers did not know the correct answer to questions about supplements from groups A, B, and C; however, with the broader analysis of the responses, it was men who got the majority of correct answers. Gajda-Konopkaand Lesiów assessed the knowledge and dietary habits of people who were active and engaged in sport. Researchers conducted a survey among 100 people aged from 18 to 30 years, leading an active lifestyle. Respondents showed theoretical knowledge of proper nutrition and its importance; however, this knowledge came mainly from the media and advice from people around them and coaches, not confirmed scientific sources. The acquired results show that people who engage in sport regularly do not choose DS inadvertently, but improperly manage water-electrolyte balance [20]. Frączek, Gacek, Grzelak undertook evaluation of the prevalence of substances that support exercise capacity among people who play sports competitively, taking into consideration gender and the nature of the discipline (endurance and mixed, e.g., strength and speed). The research included 156 people (78 women and 78 men) doing sports in various disciplines. Taking erogenous substances was declared by the vast majority of the studied athletes (86.5%), and the most widespread were vitamin and mineral formulations. Supplementation with protein and carbohydrate nutrients concerned about one-third of competitors, especially those doing gymnastics, cross-country skiing, and ski jumping. Athletes also received carnitine, BCAA, HMB, and rarely creatine, coenzyme Q10, and ginseng. The authors showed differences depending on the sex and the nature of the sport discipline practiced depending on the consumed preparations [21].

Also in the population surveyed by us, most of the respondents did not use DS, but they did know the definition of supplementation. On the other hand, the group who used DS and did not know the definition itself (73,38%) was also large. More often people who use supplementation have extent knowledge of this subject. Žeželj et al. conducted an assessment of the knowledge level and attitude regarding DS among the student population in Croatia (910 people) showing that the most physically active respondents (37.7%) took significantly more DS and had greater knowledge than people who did not use supplements [22].

Heikkilä et al. conducted knowledge research in the area of nutrition and supplements among athletes playing team games. The aim of the study was to assess knowledge among Finnish endurance athletes (156 men and 156 women, age = 17.9 ± 1.2 years) and their coaches (69 men and 25 women, age = 44.3 ± 12.3 years) [23].

Three main sports among the participants were cross-country skiing (n = 53 coaches and n = 111 competitors), orienteering (n = 13 and n = 110), and biathlon (n = 6 and n = 38). On average, the coaches (N = 94) responded to 81%  ± 9% of 79 questions about diets correctly. However, DS and nutritional recommendations for endurance athletes have turned out to be difficult. The average assessment of the nutritional skills of athletes was relatively low. Our research also shows that people who were active and having regular contact with sport could not correctly determine the caloric requirements for carbohydrates and protein with the division into types of sport activity [23].

Analyzing the correlation of the use of DS and knowledge to the age and the length of the training experience, we found that the peak of consuming the supplementation falls on the 18-24 age range, and among people with the training experience of 6-11 years appears a decision about supplementation as a support. This observation confirms the above-mentioned results of other researchers that the knowledge about the consumption and body requirements for supplements depending on the sports discipline increases with age and the duration of the training.

## 6. Limitation

A limitation of the study was the unequal distribution of the study population, with a larger proportion of female participants as compared to males.

## 7. Conclusion

High prevalence of supplement consumption was observed among the studied population; however, significant number of individuals lacked accurate information about these products. Hence, community urgently requires education and access to unbiased scientific information.

## Figures and Tables

**Table 1:** Characteristics of the study group.

	Women	Men	Total
*Age [±; SD]*	25.38 ± 5.27	25.40 ± 5.27	25.40 ± 5.27

*Weight in kg [±; SD]*	65.92 ± 11.14	65.96 ± 11.11	65.69 ± 11.11

*Height in cm [±; SD]*	171.04 ± 11.13	171.10 ± 11.11	171.10 ± 11.11

*BMI [n; *%*]*			
Underweight	24; 9.60	0; 0.00	24; 5.86
Normal	208; 83.20	133; 83.13	341; 83.17
Overweight	15; 6.00	23; 14.37	38; 9.27
Obese	3; 1.20	4; 2.50	7; 1.70

*Place of living [n; *%*]*			
Urban area	122; 48.80	124; 77.50	246; 60.00
Rural area	128; 51.20	36; 22.50	164; 40.00

*Undertaking physical activity [n; *%*]*			
Yes	145; 58.00	50; 31.25	195; 47.56
No	105; 42.00	110; 68.75	215; 52.44

**Table 2:** The knowledge about diet and supplementation with respect to sex.

*Variable*	*Men* N; %	*Woman* N; %	*Total* N; %	*p-value*
*Definition of supplementation*				
Known	71; 44.37	120; 48.00	191; 46.58	p≤0.47
Unknown	89; 55.63	130; 52.00	219; 53.42	

*Group A supplements include:*				
Bicarbonate or citrate	72; 45.00	73; 29.20	145; 35.36	
Glutamine, glucosamine	68; 42.50	114; 45.60	182; 44.39	*p≤0.001*
Ribose, melatonin	9; 5.62	36; 14.40	45; 10.99	
All correct	11; 6.88	27; 10.80	38; 9.26	

*Group B supplements include:*				
Bicarbonate or citrate	66; 41.25	88; 35.20	154; 37.56	
Glutamine, glucosamine	67; 41.87	96; 38.40	163; 39.77	p≤0.15
Vitamin E and C	22; 13.75	52; 20.84	74; 18.04	
All correct	5; 3.13	14; 5.56	19; 4.63	

*Group C supplements include:*				
Bicarbonate or citrate	33; 20.63	52; 20.80	85; 20.73	
Glutamine, glucosamine	8; 5.00	20; 8.00	28; 6.83	p≤0.32
Coenzyme Q10, nitric oxide	114; 71.25	163; 65.20	277; 67.56	
All correct	5; 3.12	15; 6.00	20; 4.88	

%* of the total caloric value of the protein in the meal is:*				
10-15%	74; 46.25	56; 22.40	130; 31.70	
10-20%	58; 36.25	118; 47.20	176; 42.93	*p≤0.001*
15-30%	25; 15.63	60; 24.00	85; 20.73	
Incorrect answer	3; 1.87	16; 6.40	19; 4.64	

%* of the total calorific value of carbohydrates in a meal is:*				
20-30%	41; 25.63	68; 27.20	109; 26.59	
40-50%	29; 18.13	75; 30.00	104; 25.37	*p≤0.001*
50-55%	85; 53.13	74; 29.60	159; 38.78	
35-45%	5; 3.11	33; 13.20	38; 9.26	

%* of the total calorific value of fats in a meal is:*				
25-30%	18; 11.25	59; 23.60	77; 18.78	
30-35%	72; 45.00	59; 23.60	131; 31.95	*p≤0.001*
15-25%	66; 41.25	113; 45.20	179; 43.66	
Incorrect answer	4; 2.50	19; 7.60	23; 5.61	

*Subjective level of knowledge about diet and supplementation in sport:*				
Lack of knowledge	6; 3.75	15; 6.00	21; 5.12	
Little knowledge	48; 30.00	119; 47.60	167; 40.73	*p≤0.001*
Average knowledge	26; 16.25	106; 42.40	132; 32.20	
Vast knowledge	80; 50.00	10; 4.00	90; 21.95	

**Table 3:** The knowledge about diet and supplementation, and undertaking physical activity.

*Variable*	*Engaged in physical activity* N; %	*Not engaged in physical activity* N; %	*Total* N; % (410)	*p-value*
*Definition of supplementation*				
Known	153; 47.52	38; 43.19	191; 46.56	p≤0.47
Unknown	169; 52.48	50; 56.81	219; 53.44	

*Group A supplements include:*				
Bicarbonate or citrate	122; 37.89	23; 26.13	145; 35.37	
Glutamine, glucosamine	148; 45.97	34; 38.64	182; 44.39	*p≤0.001*
Ribose, melatonin	29; 9.00	16; 18.18	45; 10.97	
All correct	23; 7.14	15; 17.05	38; 9.27	

*Group B supplements include:*				
Bicarbonate or citrate	126; 39.14	28; 31.81	154; 37.56	
Glutamine, glucosamine	127; 39.44	36; 40.90	163; 39.76	p≤0.28
Vitamin E and C	57; 17.70	17; 19.34	74; 18.04	
All correct	12; 3.72	7; 7.95	19; 4.61	

*Group C supplements include:*				
Bicarbonate or citrate	71; 22.04	14; 15.92	85; 20.73	
Glutamine, glucosamine	21; 6.53	7; 7.95	28; 6.83	p≤0.49
Coenzyme Q10, nitric oxide	216; 67.08	61; 69.31	277; 67.56	
All correct	14; 4.35	6; 6.82	20; 4.88	

%* of the total caloric value of the protein in the meal is:*				
10-15%	110; 34.16	20; 22.73	130; 31.71	
10-20%	136; 42.23	40; 45.45	176; 42.93	p≤0.14
15-30%	61; 18.94	24; 27.27	85; 20.73	
Incorrect answer	15; 4.67	4; 4.55	19; 4.63	

%* of the total calorific value of carbohydrates in a meal is:*				
20-30%	88; 27.33	21; 23.86	109; 26.58	
40-50%	72; 22.36	32; 36.36	104; 25.37	p≤0.001
50-55%	137; 42.55	22; 25.00	159; 38.78	
35-45%	25; 7.76	13; 14.78	38; 9.27	

%* of the total calorific value of fats in a meal is:*				
25-30%	45; 13.97	32; 36.36	77; 18.78	
30-35%	117; 36.35	14; 15.92	131; 31.95	*p≤0.001*
15-25%	142; 44.09	37; 42.04	179; 43.66	
Incorrect answer	18; 5.59	5; 5.68	23; 5.61	

*Subjective level of knowledge about diet and supplementation in sport:*				
Lack pf knowledge	12; 3.73	9; 10.22	21; 5.13	*p≤0.001*
Little knowledge	121; 37.57	46; 52.28	167; 40.73	
Average knowledge	99; 30.75	33; 37.50	132; 32.19	
Vast knowledge	90; 27.95	0; 00.00	90; 21.95	

**Table 4:** The use of DS, and activity and knowledge about diet and supplementation in sport.

*Variable*	*Use of dietary supplements* N; %	*No use of dietary supplements* N; %	*Total* N; %	*p-value*
*Definition of supplementation*				
Known	41; 26.62	150; 58.59	191; 46.58	*p≤0.001*
Unknown	113; 73.38	106; 41.41	219; 53.42	

*Group A supplements include:*				
Bicarbonate or citrate	86; 55.88	59; 23.04	145; 35.36	
Glutamine, glucosamine	40; 25.97	142; 55.47	182; 44.39	*p≤0.001*
Ribose, melatonin	16; 10.39	29; 11.33	45; 10.98	
All correct	12; 7.79	26; 10.16	38; 9.27	

*Group B supplements include:*				
Bicarbonate or citrate	36; 23.38	118; 46.09	154; 37.56	
Glutamine, glucosamine	83; 53.89	80; 31.25	163; 39.76	*p≤0.001*
Vitamin E and C	28; 18.18	46; 17.97	74; 18.05	
All correct	7; 4.55	12; 4.69	19; 4.63	

*Group C supplements include:*				
Bicarbonate or citrate	50; 32.48	35; 13.67	85; 20.73	
Glutamine, glucosamine	11; 7.14	17; 6.64	28; 6.83	*p≤0.001*
Coenzyme Q10, nitric oxide	89; 57.79	188; 73.43	277; 67.56	
All correct	4; 2.59	16; 6.26	20; 4.88	

%* of the total caloric value of the protein in the meal is:*				
10-15%	94; 61.04	36; 14.06	130; 31.71	
10-20%	32; 20.78	144; 56.25	176; 42.93	*p≤0.001*
15-30%	23; 14.94	62; 24.22	85; 20.73	
Incorrect answer	5; 3.24	14; 5.47	19; 4.63	

%* of the total calorific value of carbohydrates in a meal is:*				
20-30%	14; 9.09	95; 37.12	109; 26.58	
40-50%	29; 18.84	75; 29.29	104; 25.37	*p≤0.001*
50-55%	103; 66.88	56; 21.88	159; 38.78	
35-45%	8; 5.19	30; 11.71	38; 9.27	

%* of the total calorific value of fats in a meal is:*				
25-30%	25; 16.23	52; 20.33	77; 18.78	
30-35%	87; 56.49	44; 17.18	131; 31.95	*p≤0.001*
15-25%	38; 24.69	141; 55.07	179; 43.66	
Incorrect answer	4; 2.59	19; 7.42	23; 5.61	

*Subjective level of knowledge about diet and supplementation in sport:*				
Lack of knowledge	4; 2.60	17; 6.65	21; 5.12	
Little knowledge	22; 14.28	145; 56.64	167; 40.73	*p≤0.001*
Average knowledge	45; 29.22	87; 33.98	132; 32.20	
Vast knowledge	83; 53.90	7; 2.73	90; 21.95	

**Table 5:** The use of DS, and age and training experience.

*Variable*	*Use of dietary supplements* N; %	*No use of dietary supplements* N; %	*Total* N; %	*p- value*
*Age:*				
19-24 years old	69; 44.80	168; 65.63	237; 57.80	
25-30 years old	46; 29.87	24; 9.32	70; 17.08	*p≤0.001*
More than 31 years old	39; 25.33	64; 25.00	103; 25.12	

*Training experience:*				
1-5 years	67; 43.50	184; 71.88	251; 61.22	*p≤0.001*
6-11 years	68; 44.15	54; 21.09	122; 29.76	
12-17 years	19; 12.35	18; 7.03	37; 9.02	

**Table 6:** Knowledge about diet and supplementation in terms of undertaking physical activity.

*Variable*	*Engagement in physical activity* N; %	*No engagement in physical activity* N; %	*Total* N; %	*p-value*
*Use of dietary supplements:*				
Yes	144; 44.72	10; 11.36	154; 37.56	*p≤0.001*
No	178; 55.28	78; 88.64	256; 62.44	

*Caloric demand per kilogram of body weight in endurance sports is:*				
65 kcal/kg of body weight	69; 21.44	15; 17.04	84; 20.48	
75 kcal/kg of body weight	127; 39.44	28; 31.82	155; 37.80	p≤0.07
60 kcal/kg of body weight	40; 12.42	20; 22.73	60; 14.64	
70 kcal/kg of body weight	86; 26.70	25; 28.41	111; 27.08	

*Caloric demand per kilogram of body mass in high-speed sports is:*				
65 kcal/kg of body weight	65; 20.19	24; 27.27	89; 21.71	
75 kcal/kg of body weight	103; 31.99	18; 20.45	121; 29.52	*p≤0.001*
60 kcal/kg of body weight	117; 36.33	28; 31.82	145; 35.36	
70 kcal/kg of body weight	37; 11.49	18; 20.46	55; 13.41	

*Caloric demand per kilogram of body weight in strength sports is:*				
65 kcal/kg of body weight	62; 19.26	24; 27.27	86; 20.97	
75 kcal/kg of body weight	99; 30.74	36; 40.90	135; 32.92	*p≤0.001*
60 kcal/kg of body weight	58; 18.01	12; 13.64	70; 17.08	
70 kcal/kg of body weight	103; 31.99	16; 18.19	119; 29.03	

*Subjective level of knowledge about caloric demand per kilogram of body weight in sport with different character:*				
Lack of knowledge	12; 3.73	9; 10.23	21; 5.12	
Little knowledge	121; 37.58	46; 52.27	167; 40.73	*p≤0.001*
Average knowledge	99; 30.75	33; 37.50	132; 32.19	
Vast knowledge	90; 27.94	0; 0.00	90; 21.96	

**Table 7:** Training experience and knowledge about diet and supplementation in sport.

*Variable*	*Training experience* *1-5 years:* N; %	*Training experience* *6-11 years:* N; %	*Training experience* *12-17 years:* N; %	*Total* N; %	*p-value*
*Definition of supplementation*					
Known	111; 44.22	58; 47.54	22; 59.46	191; 46.59	p≤0.21
Unknown	140; 55.78	64; 52.46	15; 40;54	219; 53.41	

*Group A supplements include:*					
Bicarbonate or citrate	79; 31.47	49; 40.18	17; 45.95	145; 35.37	
Glutamine, glucosamine	102; 40.64	61; 50.00	19; 51.35	182; 44.39	*p≤0.001*
Ribose, melatonin	37; 14.74	8; 6.55	0; 00.00	45; 10.97	
All correct	33; 13.15	4; 3.27	1; 2.70	38; 9.27	

*Group B supplements include:*					
Bicarbonate or citrate	76; 30.29	56; 45.92	22; 59.47	154; 37.56	
Glutamine, glucosamine	98; 39.04	53; 43.44	12; 32.43	163; 39.77	*p≤0.001*
Vitamin E and C	60; 23.90	11; 9.01	3; 8.10	74; 18.04	
All correct	17; 6.77	2; 1.63	0; 00.00	19; 4.63	

*Group C supplements include:*					
Bicarbonate or citrate	50; 19.92	31; 25.42	4; 10.81	85; 20.73	*p≤0.001*
Glutamine, glucosamine	20; 7.97	8; 6.55	0; 00.00	28; 6.83	
Coenzyme Q10, nitric oxide	164; 65.34	80; 65.57	33; 89.19	277; 67.56	
All correct	17; 6.77	3; 2.46	0; 00.00	20; 4.88	

%* of the total caloric value of the protein in the meal is:*					
10-15%	61; 24.31	54; 44.27	15; 40.54	130; 31.70	
10-20%	99; 39.44	56; 45.90	21; 56.76	176; 42.94	*p≤0.001*
15-30%	76; 30.27	8; 6.55	1; 2.70	85; 20.73	
Incorrect answer	15; 5.98	4; 3.28	0; 00.00	19; 4.63	

%* of the total calorific value of carbohydrates in a meal is:*					
20-30%	57; 22.73	34; 27.87	18; 48.65	109; 26.58	*p≤0.001*
40-50%	78; 31.07	23; 18.85	3; 8.10	104; 25.37	
50-55%	82; 32.66	61; 50.00	16; 43.25	159; 38.78	
35-45%	34; 13.54	4; 3.28	0; 00.00	38; 9.27	

%* of the total calorific value of fats in a meal is:*					
25-30%	68; 27.10	8; 6.56	1; 2.70	77; 18.79	
30-35%	59; 23.50	53; 43.44	19; 51.35	131; 31.95	*p≤0.001*
15-25%	105; 41.83	58; 47.54	16; 43.25	179; 43.66	
Incorrect answer	19; 7.57	3; 2.46	1; 2.70	23; 5.60	

*Subjective level of knowledge about diet and supplementation in sport:*					
Lack of knowledge	20; 7.98	1; 0.82	0; 00.00	21; 5.13	
Little knowledge	100; 39.84	50; 40.98	17; 45.95	167; 40.73	*p≤0.001*
Average knowledge	115; 45.81	12; 9.84	5; 13.51	132; 32.19	
Vast knowledge	16; 6.37	59; 48.36	15; 40.54	90; 21.95	

**Table 8:** Age and knowledge about diet and supplementation in sport.

*Variable*	*Age* *19-24 years* N; %	*Age* *25-30 years* N; %	*Age more than 31 years* N; %	*Total* N; %	*p-value*
*Definition of supplementation*					
Known	108; 45.56	22; 28.57	61; 59.22	191; 46.58	*p≤0.001*
Unknown	129; 54.44	48; 62.33	42; 40.78	219; 53.52	

*Group A supplements include:*					
Bicarbonate or citrate	70; 29.55	36; 51.43	39; 37.87	145; 35.36	
Glutamine, glucosamine	97; 40.92	25; 35.71	60; 58.25	182; 44.39	
Ribose, melatonin	37; 15.61	5; 7.14	3; 2.91	45; 10.98	*p≤0.001*
All correct	33; 13.92	4; 5.72	1; 0.97	38; 9.27	

*Group B supplements include:*					
Bicarbonate or citrate	72; 30.39	22; 31.43	60; 58.25	154; 37.56	
Glutamine, glucosamine	88; 37.13	38; 54.29	37; 35.82	163; 39.77	
Vitamin E and C	61; 25.73	8; 11.43	5; 4.85	74; 18.04	*p≤0.001*
All correct	16; 6.75	2; 2.85	1; 0.97	19; 4.63	

*Group C supplements include:*					
Bicarbonate or citrate	56; 23.63	9; 12.86	20; 19.42	85; 20.73	
Glutamine, glucosamine	23; 9.70	2; 2.85	3; 2.91	28; 6.83	
Coenzyme Q10, nitric oxide	142; 59.92	56; 80.00	79; 76.70	277; 67.56	*p≤0.001*
All correct	16; 6.75	3; 4.29	1; 0.97	20; 4.88	

%* of the total caloric value of the protein in the meal is:*					
10-15%	52; 21.94	40; 57.15	38; 36.90	130; 31.70	
10-20%	93; 39.24	18; 25.71	65; 63.10	176; 42.92	*p≤0.001*
15-30%	75; 31.65	10; 14.29	0; 00.00	85; 20.73	
Incorrect answer	17; 7.17	2; 2.85	0; 00.00	19; 4.65	

%* of the total calorific value of carbohydrates in a meal is:*					
20-30%	55; 23.21	12; 17.14	42; 40.78	109; 26.59	
40-50%	74; 31.22	10; 14.29	20; 19.42	104; 25.36	*p≤0.001*
50-55%	76; 32.06	42; 60.00	41; 39.80	159; 38.78	
35-45%	32; 13.51	6; 8.57	0; 00.00	38; 9.27	

%* of the total calorific value of fats in a meal is:*					
25-30%	64; 27.00	5; 7.14	8; 7.78	77; 18.79	
30-35%	54; 22.78	41; 58.57	36; 34.95	131; 31.95	*p≤0.001*
15-25%	101; 42.63	21; 30.00	57; 55.33	179; 43.66	
Incorrect answer	18; 7.59	3; 4.29	2; 1.94	23; 5.61	

*Subjective level of knowledge about diet and supplementation in sport:*					
Lack of knowledge	18; 7.59	1; 1.44	2; 1.94	21; 5.13	
Little knowledge	97; 40.93	17; 24.28	53; 51.45	167; 40.73	*p≤0.001*
Average knowledge	111; 46.84	13; 18.57	8; 7.78	132; 32.19	
Vast knowledge	11; 4.64	39; 55.71	40; 38.83	90; 21.95	

## Data Availability

The results of our study supporting the findings are included within this paper. In order to protect patient privacy, the personal data supporting the findings of this study are restricted by the Bioethics Committee at the University of Rzeszów. Data are available to researchers who meet the criteria for access to confidential data from the corresponding author.
